# Functionalized chitosan as nano-delivery platform for CRISPR-Cas9 in cancer treatment

**DOI:** 10.1016/j.ajps.2025.101041

**Published:** 2025-02-26

**Authors:** Asif Nawaz, Nur Syamimi Ariffin, Tin Wui Wong

**Affiliations:** a Advanced Drug Delivery Laboratory, Gomal Centre of Pharmaceutical Sciences, Faculty of Pharmacy, Gomal University, DIKhan 29050, Pakistan; b Department of Pharmacology and Life Sciences, Faculty of Pharmacy, Universiti Teknologi MARA, Puncak Alam 42300, Malaysia; c Particle Design Research Group, Faculty of Pharmacy, Universiti Teknologi MARA, Puncak Alam 42300, Malaysia; d Non-Destructive Biomedical and Pharmaceutical Research Centre, Smart Manufacturing Research Institute, Universiti Teknologi MARA, Puncak Alam 42300, Malaysia; e Department of Industrial Pharmacy, Faculty of Pharmacy, Silpakorn University, Nakhon Pathom, 73000, Thailand

**Keywords:** Cancer, Chitosan, CRISPR-Cas9, Excipient, Nanocarrier

## Abstract

CRISPR-Cas system permanently deletes any harmful gene-of-interest to combat cancer growth. Chitosan (CS) is a potential cancer therapeutic that mediates via PI3K/Akt/mTOR, MAPK and NF-kβ signaling pathway modulation. CS and its covalent derivatives have been designed as nanocarrier of CRISPR-Cas9 alone (plasmid or ribonucleoprotein) or in combination with chemical drug for cancer treatment. The nanocarrier was functionalized with polyethylene glycol (PEG), targeting ligand, cell penetrating ligand and its inherent positive zeta potential to mitigate premature clearance and particulate aggregation, and promote cancer cell/nucleus targeting and permeabilization to enable CRISPR-Cas9 acting on the host DNA. Different physicochemical attributes are required for the CS-based nanocarrier to survive from the administration site, through the systemic circulation-extracellular matrix-mucus-mucosa axis, to the nucleus target. CRISPR-Cas9 delivery is met with heterogeneous uptake by the cancer cells. Choice of excipients such as targeting ligand and PEG may be inappropriate due to lacking overexpressed cancer receptor or availability of excessive metabolizing enzyme and immunoglobulin that defies the survival and action of these excipients rendering nanocarrier fails to reach the target site. Cancer omics analysis should be implied to select excipients which meet the pathophysiological needs, and chitosan nanocarrier with a “transformative physicochemical behavior” is essential to succeed CRISPR-Cas9 delivery.

## Graphical abstract

**Figure:**
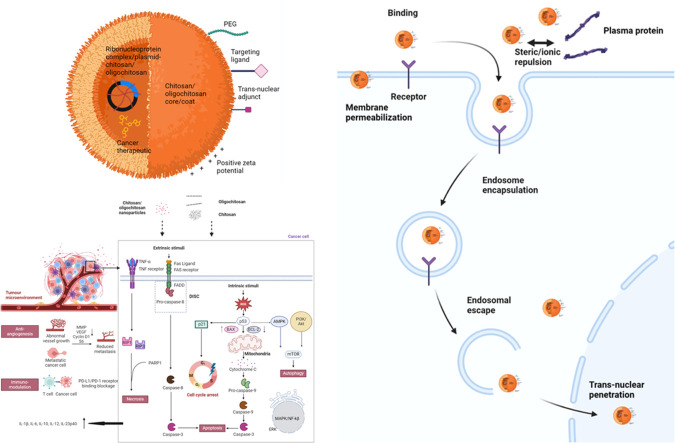

## 1 Overview of CRISPR-Cas9

RNA interference (RNAi)-mediated knockdown is frequently used to deplete cells of a protein-of-interest [1]. However, since 2012, a RNA-based regulatory system that emerges from a relatively unexplored bacteria world, namely CRISPR-Cas (clustered regularly interspaced short palindromic repeats-associated protein), has been introduced to the field of genetic engineering. CRISPR-Cas is a genome editing technology engineered based on a natural system used by bacteria to protect against infection of viruses, bacteriophages and plasmids [2,3]. Compared to RNAi that aims at the RNA expression, CRISPR-Cas is a powerful tool that allows researchers to edit a genomic DNA of an organism by using a single guide RNA (sgRNA) to target any gene-of-interest with high accuracy and selectivity.

In contrast to using RNAi that targets the RNA expression, the CRISPR-Cas technique deletes the expression of a gene permanently by targeting a genomic locus either on the first or any exon that will result in the knockout of protein expression as well as its functions [4]. CRISPR technology enables a complete elimination of a gene from cells by shifting the reading frame of the gene in comparison to RNAi system that depletes protein expression as a result of post-transcriptional down-regulation without changing the genetic code of the gene. CRISPR-Cas system permanently deletes the expression of a gene from a cellular system and the related protein that determines the phenotype of cells.

**Fig. 1:**
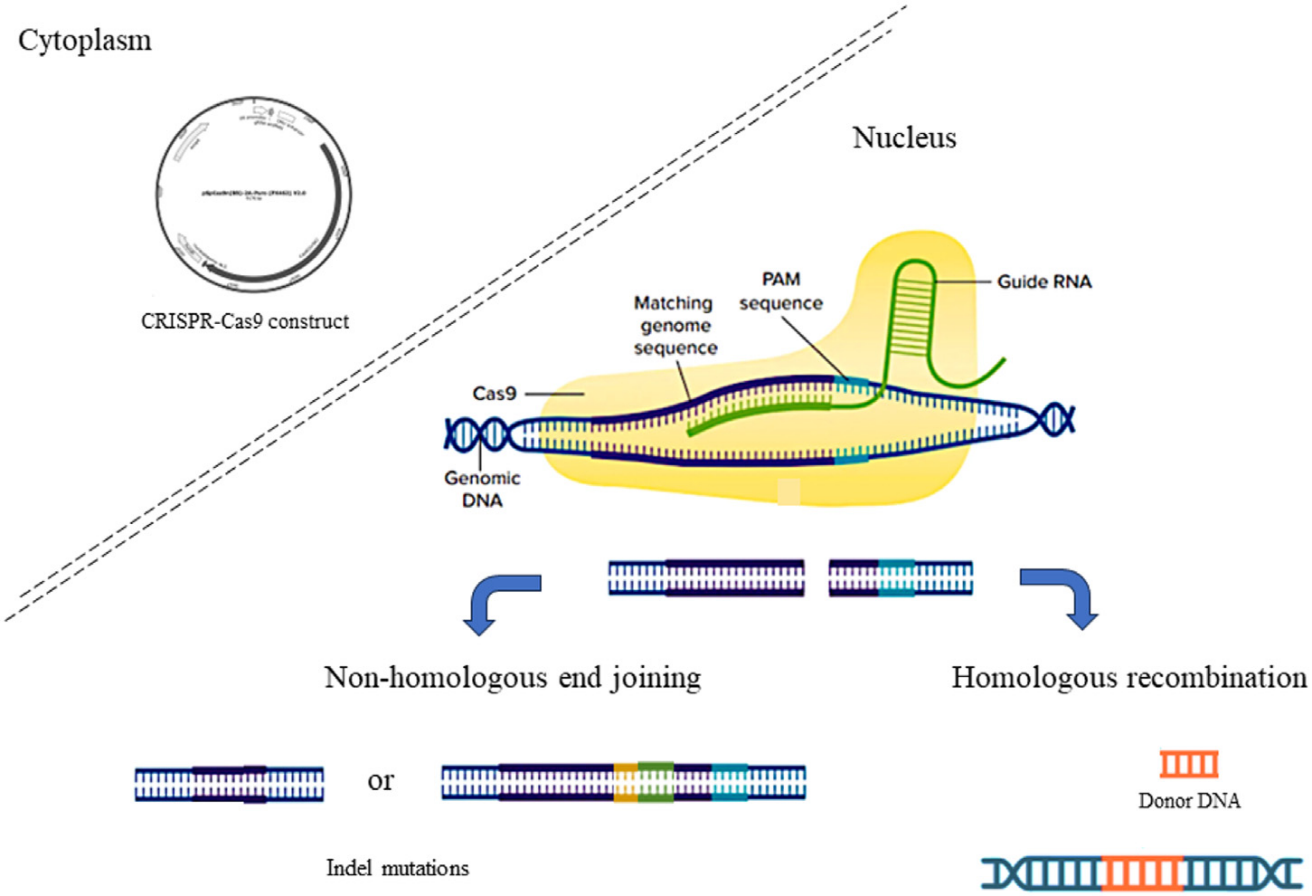
CRISPR-Cas9 and its mechanism of gene editing.

The CRISPR-Cas system can be categorized into two classes based on the structures and functions of the Cas proteins. The first category is Class I (type I, III and IV) whereas the second category is Class II (type II, V and VI) [5,6]. A Cas protein complex of class I consists of multiple subunits, whereas the Cas protein complex of class II contains only a single subunit. Genetic engineering uses type II CRISPR-Cas9 extensively due to its simple structure [5]. The CRISPR-Cas9 system is constituted of two essential components: guide RNA (gRNA) and CRISPR-associated proteins (Cas9) [7] (Fig. 1). In the first genome-editing experiment, the Cas9 protein was extracted from *Streptococcus pyogenes* (SpCas-9) [8]. Cas9 is a DNA endonuclease with a large (1,368 amino acids) multi-domain structure that cleaves target DNA into double-stranded breaks [9]. The gRNA is comprised of two parts, the CRISPR RNA (crRNA) and the trans-activating CRISPR RNA (trcrRNA). trcrRNA is a long string of loops that serves as a scaffold for Cas9 nuclease, while crRNA is an 18–20 base pair length molecule that identifies the target sequence via pairing. In order to target almost any gene sequence intended for editing, crRNA and trcrRNA can be synthesized to form a sgRNA as the gene-editing tool [10]. There are two major regions in Cas9, namely the recognition (REC) lobe and the nuclease (NUC) lobe [11]. There are two REC lobes, one for binding gRNA and one for interacting with protospacer adjacent motif (PAM). The NUC lobe consists of RuvC, HNH, and PAM domains. PAM interacting domains confer PAM specificity and initiate binding to target DNA, while RuvC and HNH domains cut single-stranded DNA [12].

Transfecting CRISPR-Cas9 into the nucleus of a host cell is accompanied by its locking onto a 5′-NGG sequence (PAM) and gRNA-target DNA base pairing [13]. Subsequently, the Cas9 will unzip a DNA double strand before cutting it at the third base pair upstream of the PAM sequence [14,15] (Fig. 1). A DNA repair mechanism will be taking place to amend the DNA damage through a non-homologous end-joining (NHEJ) or homology-directed repair (HDR). The NHEJ repair process is relatively random in nature and error prone, and it leads to an insertion or a deletion of DNA nucleotides, leading to genomic mutations of various lengths. This will disrupt the reading frame of a coding sequence, resulting in a loss or gain of protein function. HDR will introduce a copy of DNA carrying a desired sequence where random mutations can be avoided during the repair process. Specifically, in HDR, by the time DNA is cut by the Cas9 nuclease, the DNA template that is introduced will form complement with the cut ends, hence replacing the original sequence with a new piece of DNA [16].

## 2 Cancer and CRISPR-Cas9

Nearly 19.3 million new cancer cases and almost 10 million cancer deaths have been estimated in 2020 with an expected rise of new cases to 28.4 million in 2040 globally [17]. Female breast, lung, colorectum, prostate and stomach cancers are among the most common variants diagnosed. Lung cancer has the highest mortality incidence (18 %), followed by colorectum (9.4 %), liver (8.3 %), stomach (7.7 %) and breast (6.9 %) cancers. Cancer is characterized by an abnormal proliferation of cells with the capability to metastasize through circulatory and lymphatic systems [18]. There is increasing evidence which suggests that genetic mutations are critically responsible for cancer pathogenesis and growth under multiple epigenetic and genetic conditions [19]. Chemotherapy is the standard treatment mode for both primary and metastatic cancers via the induction of cancer apoptosis or necrosis [20]. Conventional chemotherapy brings about adverse drug effects due to non-selective action of chemotherapeutics to normal cells [[21], [22], [23]]. In combination with multiple drug resistance and poor aqueous drug solubilitythat negate cancer cell permeation and interaction, these render a high failure rate of cancer treatment [24]. Developing a more efficient and safer cancer treatment approach is critical to resolve such hurdles. Using exogenous nucleic acids, gene therapy can substitute, replace or suppress the malfunction gene [25]. Through disrupting messenger RNA (mRNA) stability or triggering mRNA degradation, the small interfering RNA (siRNA) and microRNA (miRNA) can suppress cancer-related gene expression. However, these gene suppression effects are transitory and may have risks of off-target effects. Unlike conventional chemotherapeutics and gene therapy, CRISPR-Cas9 offers a better option where it is envisaged to edit any harmful gene-of-interest in cancer patients in involving metastasis and drug resistance development [[26], [27], [28]]. It is capable of permanently knocking out unwanted phenotypes as part of a strategy to combat cancer growth.

Using CRISPR-Cas9, the desired DNA sequence can be introduced in cancer patients to alter their genetic code to meet the treatment goal [29]. Early attempt successfully reverses the malignancies of hereditary tyrosinemia in liver cells of mice with a mutated *Fah* gene [30]. The CRISPR-Cas9 system has also been demonstrated to suppress the *MLL3* gene from inducing acute myeloid leukemia in *ex vivo* studies using mice [31]. It provides a synergistic improvement in head and neck cancer treatment using epirubicin by eliminating the *HuR* gene that is responsible for inducing cancer cell survival, invasion and resistance [32]. Intriguingly, T cells can be engineered with chimeric antigen receptor (CAR) to treat cancer [33]. CAR can activate T cells to recognize specific tumor antigen and destroy the cancerous tissue [34]. T cell targeting CD19 in leukaemia patients is functional with a durable complete cancer remission due to the expression of such marker is found only in B cells [35]. CAR-T therapy is workable against solid tumors and its efficiency is strengthened with the advance of CRISPR-Cas9 system to knock out endogenous genes such as major histocompatibility complexes (MHCs) in order to allow the development of universal CAR-T cells or knock in functional genes such as natural killer genes and interleukins to produce a more comprehensive CAR-T cells’ microenvironment [[36], [37], [38]].

## 3 Nano delivery technology

Genome editing technology is a rapidly expanding sector for the treatment of both acquired and hereditary disorders including cancer [29,39]. Effective delivery of CRISPR-Cas9 to the intended cancer tissues and cells is nevertheless met with absorption and transport barriers from the site of administration to the target nucleus [40]. Two distinctive vectors, viral and non-viral, have been adapted for CRISPR-Cas9 delivery [41]. Viral vector is the most widely used approach [42]. It involves integrating CRISPR-Cas9-encoding sequences into the viral genome and releasing the CRISPR-Cas9 gene complex into infected cells with high levels of transfection efficiency and gene expression. Viral vector however is characterized by minimal gene loading capacity, and it may trigger unexpected host immunological reactions and disastrous inflammation [43,44].

A material or an end product, that is designed to have at least one external dimension or an internal or surface structure in the range of about 1 to 100 nm or exhibits properties or phenomena that are attributable to a dimension up to 1,000 nm, is considered under the purview of nanotechnology in accordance with the United States Food and Drug Administration [45]. Nanoparticles (NPs) or submicron particles of 1 to 1,000 nm in size have been adopted to encapsulate, stabilize and target delivery of various chemotherapeutics at organ, tissue, cell and organelle levels with the primary aim to improve drug efficacy and minimize adverse off-target biological responses [23,46]. Through judicious choice of excipient, formulation and dosage form design strategy, the efficiency of therapeutic encapsulation, stabilization and target delivery can be raised via an interplay of nanocomposition and chemistry as well as NP size, polydispersity index, zeta potential, shape, surface roughness, crystallinity and hardness [23,[47], [48], [49], [50]]. With reference to gene and protein delivery, mesoporous silica nanoparticles [[51], [52], [53], [54], [55]], non-porous silica nanoparticles [56], metal-organic frameworks (zirconium-fumaric acid, aluminium trimeric cluster- triaminotrinitrobenzene, zinc-2-methylimidazole) [[57], [58], [59]], polymeric NPs [polyallylamine-thiol-PEG, PEG-b-poly(trimethylene carbonate-co-dithiolane trimethylene carbonate)-b-polyethylenimine, polylactic-co-glycolic acid (PLGA)-poly(3-carbobenzoxyl-l-lysine)-PEG, polyethylenimine-hydrazide, chitosan-m-PEG] [[60], [61], [62], [63], [64]], nanogels [hyaluronic acid (HA), dendritic polyglycerol-2-formylphenylboronic acid-citraconic anhydride, cholesterol-bearing pullulan, PLGA] [[65], [66], [67], [68]], micelle (PEG-poly[N-[N′-(2-aminoethyl)−2-aminoethyl]aspartamide], PEG-b-poly(aminopalmitic acid)-b-poly(aspartic acid)) [69,70], phenylboronic acid dendrimer [71], and lipid NPs (1,2-dioleoyl-sn glycero‑3-phosphoethanolamine, 1,2-di-O-octadecenyl-3 trimethylammonium propane, 1,2-dipalmitoylsnglycero-3-phosphoethanolamine N-[methoxy(PEG)−2000], β-l-arginyl-2,3-l-diaminopropionic acid-N-palmityl-N-oleylamide trihydrochloride, 1,2-dilinoleyloxy-3-dimethylaminopropane, 2,2-dilinoleyl-4-dimethylaminomethyl-[1,3]-dioxolane, 2,2-dilinoleyl-4-(2-dimethylaminoethyl)-[1,3]-dioxolane, α-tocopherol) [[72], [73], [74], [75]] have been developed over the past 20 years. NPs as therapeutic carrier increase transfection effectiveness, decrease off-target delivery, minimize immunological and systemic adverse effects and reduce toxicities associated with viral vectors [[76], [77], [78], [79]].

Nano delivery systems such as liposomes, lipid NPs, polymeric NPs and gold NPs have been formulated for CRISPR-Cas9 delivery by means of strategies adopted by gene and protein therapeutics [80,81] (Table 1). Specifically, the negative charges of CRISPR plasmids, mRNAs, or gRNAs have been exploited to complex these therapeutics with the cationic nanocarriers. Hydrophobic (lipid), cationic (protamine, polyethyleneimine, polydopamine, poly (β-amino ester), dendrimer, DOPE, DOTAP) and cell penetrating peptide (TAT) are excipients introduced to promote the transmembrane transport of CRISPR-Cas9. Lipids are an effective delivery vehicle where Lipofectamine and RNAiMAX are introduced commercially to deliver CRISPR-Cas9 in cancer treatment. Using lipid NPs, it was found that 80 % of sgRNA/Cas9 ribonucleoprotein (RNP) are delivered into human cells, giving rise to 80 % gene modification [82]. Exosomes, on the other note, can act as the carrier of CRISPR-Cas9 and transport them through the blood-brain barrier overcoming the main drawbacks associated with viral and non-viral vectors [83]. Different types of exosomes such as cancer-derived exosomes and engineered exosomes are developed for the delivery of CRISPR-Cas9 [44,84]. Tumor exosomes may however harbor cancer cell-derived genomic materials such as long non-coding RNAs (lncRNA) that can elicit neoplastic effects in target cells. Overall, delivery by NPs favors cellular uptake of high molecular weight (MW, 163 kDa) CRISPR-Cas9 complex via endocytosis and its penetration into nucleus with reduced systemic degradation and instigation of immunogenicity [[85], [86], [87]].

**Table 1:** Examples of nanocarriers of CRISPR/Cas9.

Delivery vehicle	Cargo	Size/Zeta potential	Cell line/ *In vivo*	Target site	Remark	Ref
CLAN	mCas9 and gRNA	130 nm 31.5 mV	Primary BMDMs C57BL/6 mice IV	NLRP3	CRISPR-Cas9 was delivered into macrophages to treat NLRP3-dependent inflammatory diseases. Macrophages underwent efficient gene editing up to 53 %. NLRP3 gene of macrophages was disrupted in C57BL/6 mice.	[88]
LHNPs	CRISPR-Cas9 plasmid	95 nm -	U87 cells Mice IV	PLK1	LHNPs is a versatile tool for CRISPR-Cas9 delivery. LHNPs can be adopted to modulate cancer biology *in vitro* and *in vivo* as a gene therapy.	[89]
Exosome–liposome hybrid NPs	CRISPR-Cas9 plasmid	100 nm -	MSCs C57BL/6 mice	mRunx2 hCTNNB1	Using exosome-liposome hybrid NPs, CRISPR-Cas9 can be successfully delivered to MSCs. *In vivo* studies show gene manipulation can take place using exosome liposome hybrid NPs.	[90]
CLAN	CRISPR-Cas9 plasmid	100 nm 18.5 ± 2.8 mV	BMDMs; C57BL/6 mice; IV	Ntn1	CLAN carrying the CRISPR-Cas9 plasmid can specifically express Cas9 in macrophages and monocyte precursors, resulting in 30 % gene knockouts *in vitro* and 20 % *in vivo*.	[91]
PCNP	CRISPR-Cas9 plasmid	200 nm -	HeLa cells	RHBDF1	PCNP expressed efficient genome editing.	[92]
Polymer (PS)/inorganic (CaCO_3_ and CaP) hybrid NPs	CRISPR-Cas9 plasmid	327 ± 13 nm 8.49 ± 0.20 mV	MCF-7 cells	CDK11	*In situ* detection of proteins and effective genome editing were attainable using a single NP system.	[93]
Gold nanocluster/lipid core-shell nanocarrier	CRISPR-Cas9 plasmid and sgRNA	103.7 ± 3.8 nm 35.2 ± 5.6 mV	A375 cells; BALB/c nude mice; IV	PLK1	*In vivo* cancer PLK1 genome-editing induced approximately 75 % suppression of the melanoma progression.	[94]
Amphiphilic penetrating peptide NPs	CRISPR-Cas9 plasmid	220 nm −17 mV	Magi CD4^+^/CCR5^+^ cells	CCR5	Two viral peptides, mimicking the human immunodeficiency virus and simian virus 40, with cell/nucleus-targeting capabilities were co-assembled in their active conformations into well-defined nanoparticulate carrier. These viral-like NPs can penetrate cellular membrane and deliver genetic therapeutic into the nucleus.	[95]
TAT peptide-modified lipid (DOTAP, DOPE, cholesterol) encapsulated gold NPs	CRISPR-Cas9 plasmid	101.2 ± 5.6 nm 36.4 mV	A375 cells; A375 bearing BALB/c nude mice; IV	PLK1	Efficient CRISPR-Cas9 delivery and targeted gene editing were effected. PLK1 protein was downregulated by nearly 65 % in A375 cells.	[96]
Self-assembled DNA nanoclews made of NC-12, coated with polyethyleneimine	CRISPR-Cas9 plasmidand sgRNA	56 nm 18.6 ± 4.1 mV	U2OS-EGFP cells; U2OS.EGFP bearing mice; IT	U2OS-EGFP	∼25 % of the U2OS-EGFP cells in the frozen tumor sections near the site of injection showed no EGFP expression in the mice.	[97]
Hyperbranched cationic PBAEs nanocapsules	CRISPR-Cas9 RNP and sgRNA	200 nm Neutral	CT-2A cells, HEK-293T cells, B16-F10 cells; MSC-083 C57BL/6 J mice; IT	GFP Human CXCR4 gene	A carboxylate-linked polymer with moderate hydrophobicity was best suited for cellular internalization and endosome disruption in cells. Using carboxylate-liganded polymers, CRISPR gene editing was triggered by Cas9-RNPs (160 kDa). Almost 80 % of GFP knockouts were achieved *in vitro*. In comparison with commercial reagents such as Lipofectamine, CRISPRMax or jet CRISPR, the PBAEs nanocapsules provided higher levels of gene editing (16 % vs 43 %). Further, the HEK-293T cells were able to undergo 4 % homology-directed repair with PBAEs nanocapsules targeting the human CXCR4 gene. PBAE nanocapsules, intra-cranially injected into a mouse glioma model, resulted in CRISPR gene editing.	[98]
PBA dendrimer	CRISPR-Cas9 RNP and sgRNA	300 nm 38.3 mV	HeLa cells	CTNNB1	PBA-rich cationic polymer could bind CRISPR-Cas9 RNP into stable NPs and allow efficient cytosolic delivery and genome editing.	[71]
Polydopamine NPs	CRISPR-Cas9 plasmid and sgRNA	85 nm -	B16F10cells	PD-L1	pHCP and natural resveratrol can be co-delivered to achieve permanent genome editing of PD-L1 as well as reprogramming of immune suppressive tumor microenvironment through PD-L1-mediated glucose metabolism. NIF-II light was used to mediate a photothermal effect that not only caused the transcription of sgRNA targeting PD-L1 and Cas9, but also induced the release of resveratrol, which led to immunogenic death in cancer cells through stimulation of anti-tumor immune responses.	[99]
Arginine-functionalized gold NPs	CRISPR-Cas9 RNP and sgRNA	- -	HeLa cells	–	Promising nuclear and cytoplasmic delivery of CRISPR-Cas9 protein up to 90 %, with a genome editing efficiency of 23 %–30 %.	[100]
Cationic nanoliposome	CRISPR-Cas9 plasmidand sgRNA	145.7 ± 2.6 nm 51.33 ± 1.8 mV	HPV 16-positive SiHa cervical cancer cell; Female nude mice; IT	HRHPV16 E6/E7 oncogenes	An effective CRISPR-Cas9 system to inhibit the proliferation of HPV16-positive cervical cancer SiHa cells and to induce apoptosis by inactivating the HRHPV16E6/E7 oncogenes. A significant reduction in tumor growth was observed in nude mice with no significant toxicity. The cationic liposome was a pH-sensitive complex that was stable under physiological conditions but was disassembled in cancerous tissue to exert cytotoxic actions in response to acidic environment.	[101]
Protamine-gold NPs	CRISPR-Cas9 plasmidand sgRNA	200 nm 35 mV	HeLa cells	HPV18E7 oncogene	Protamine-gold NPs were able to target nuclei and were resistant to protease degradation. This system can effectively knock out the oncogenic E7 gene, which suppressed the proliferation of HeLa cells, validating the successful use of CRISPR-Cas9-based gene editing as therapeutics.	[102]

Abbreviations: CLAN, cationic lipid-assisted nanoparticles; BMDMs, Bone marrow-derived macrophages; mCas9, Cas9 mRNA; gRNA, guide RNA; NLRP3, NOD-, LRR- and pyrin domain-containing protein 3; MSCs, mesenchymal stem cells; LHNPs, liposome-templated hydrogel nanoparticles; mRunx2, CRISPR interference system targets gene; hCTNNB1, CRISPR cleavage system targets hCTNNB1gene; PLK1 polo-like kinase 1; Ntn1, Netrin-1; PCNP, cationic polyethyleneimine-β-cyclodextrin nanoparticles; PS, Protamine sulfate; CaCO_3,_ calcium carbonate; CaP, calcium phosphate; Hemoglobin subunit beta, RHBDF1, rhomboid 5 homolog 1; CDK11, cyclin-dependent kinase 11; CCR5, chemokine receptor; NC-12, Twelve nucleotides complementary to the 5′-end of the sgRNA; pHCP, CRISPR-Cas9 plasmid with heat-inducible promoters; NIF-II, near infrared-II; PBAEs, poly (β-amino ester)s; GFP, green fluorescent protein; CXCR4, Human chemokine receptor type 4; PBA, phenylboronic acid; AAVS1, adeno-associated virus integration site 1; HBB, hemoglobin subunit beta; CTNNB1, catenin beta-1; HPV, human papillomavirus; HRHPV16 E6/E7 oncogenes, high-risk human papillomaviral 16 E6 and E7 oncogenes; HPV18E7 oncogene, human papillomavirus type 18 E7 oncogene; DOTAP, 1,2-dioleoyl-3-trimethylammonium propane; DOPE, Dioleoylphosphatidylethanolamine; EGFP, enhanced green fluorescent protein; IT, intra-thecal; IV, intra-venous.

Cells: Human brain cancer U87 cells; Cervical cancer HeLa cells; Human breast cancer MCF-7 cells; Human melanoma A375 cells; Human osteosarcoma U2OS-EGFP cells; Human embryonic kidney HEK-293T cells; Murine melanoma B16-F10 cells; Primary human adipose-derived MSC-083 mesenchymal stem cells; Melanoma B16F10cells.

## 4 Chitosan (CS) as nanovehicle of CRISPR-Cas9

### 4.1 Physicochemical basis

Chitin is the second most abundant polysaccharide in nature after cellulose [[103], [104], [105]]. It is constituted of a β-(1–4) linked linear cationic heteropolymer consisting of 2-acetamide-2-deoxy-d-glucopyranose (N–acetyl–D–glucosamine) and randomly distributed units of 2-amino-2-deoxy-D glucopyranose (D–glucosamine). Chitin is found in exoskeleton building material of marine crustacean and insects, cell wall of fungi and microorganisms [106]. It is characterized by a degree of deacetylation less than 10 % with MWs as high as 2,500 kDa along with a degree of polymerization of 5,000–10,000 monomeric residues [50]. It is relatively water-insoluble and as such has been subjected to deacetylation and/or MW reduction into acid-soluble CS by means of alkaline hydrolysis or enzymatic deacetylation using chitinases and chitosanases [[107], [108], [109], [110], [111], [112]]. Harsher chemical (*i.e.* sodium hydroxide, hydrogen peroxide) or chitooligosaccharide deacetylase treatment can be adopted to further degrade the CS into water-soluble oligochitosan. The oligochitosan is characterized by a degree of polymerization ranges from only 2 to 20 units per segment giving them MWs below 5 kDa.

CS including oligochitosan has been employed in design and development of drug delivery system from macro-to-nano scale owing to its biodegradable, biocompatible and non-allergenic attributes [[113], [114], [115], [116]]. Its widespread application is attributed to matrices made of CS can have their aqueous solubility, viscosity, drug encapsulation, drug release, mucoadhesiveness and mucosal permeation properties modulated to meet the drug delivery specification as a function of MW, degree of deacetylation, polymer content and covalent modification given that the CS possesses amino and hydroxyl functional groups for graft conjugation [23,114,117,118]. Further, CS being a cationic polysaccharide with repeating units of N-acetylglucosamine and d-glucosamine is well known for its anti-bacterial, anti-fungal, anti-viral, anti-inflammatory, anti-oxidant, anti-diabetic, anti-cancer and wound healing activities that could possibly synergize the biological effects of therapeutical agents [23,104,114,[119], [120], [121]]. Lower MW and highly deacetylated CS has a greater affinity for aqueous medium as smaller molecules that are rich in amino group favor CS chain protonation which develops inter-chain repulsion and attracts the surrounding water molecules to hydrate the CS [122,123]. The dissolved CS exhibits an increase in capacity to interact with the negatively charged lipid and/or protein domains of the cellular membrane leading to reorganization of the membrane permeability that promotes its anti-microbial, anti-oxidant and anti-cancer activities.

### 4.2 Anti-cancer activity

**Fig. 2:**
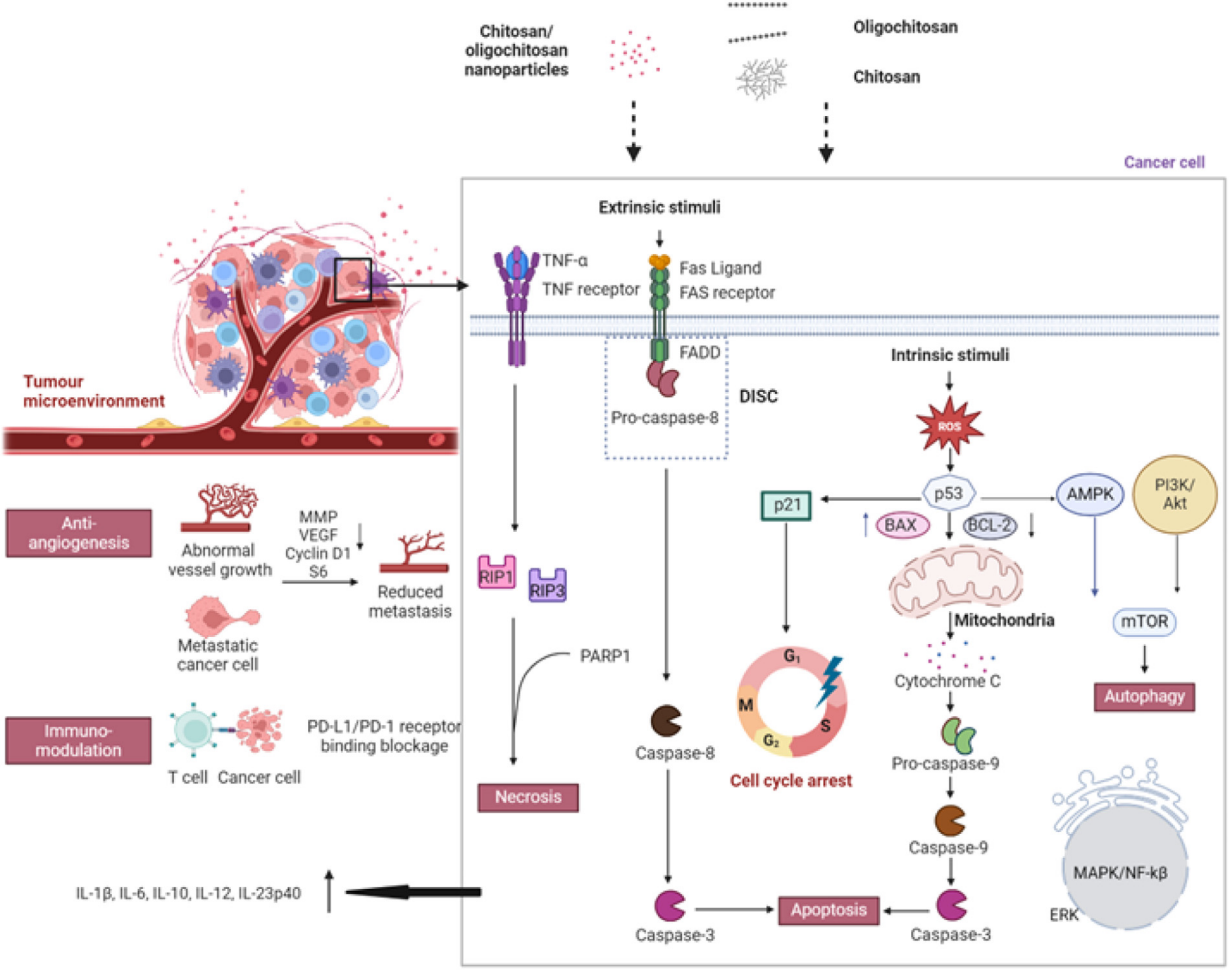
Cancer biology of CS/oligochitosan and their NPs (prepared by Biorender).

CS has lately been advocated as a potential cancer therapeutic (Fig, 2). The low MW CS (50–190 kDa) and oligochitosan are more cancer toxic *in vitro* and *in vivo* than the higher MW CS (> 190 kDa) [109]. Strong positively and negatively charged CS or carboxymethyl derivatives are more active against cancer than the neutral molecules. The positively charged CS is deemed able to be attracted to cancer cells and stromal cells in the tumor microenvironment, whereas the negatively charged CS may fluidize the cancer cell membrane through the charge-charge repulsion thereby enabling its intake into the cytoplasmic compartment. The association of CS with the cancer surface receptor and/or intracellular sub-organelles is translated to apoptosis/necrosis/autophagy, G_0_/G_1_/S phase cell cycle arrest, immuno-responses, and mitigation of angiogenic and metastasis tendency of cancer cells involving the suppression of phosphoinositide 3-kinases (PI3K)/protein kinase B (AKT)/mammalian target of rapamycin (mTOR), mitogen-activated protein kinase (MAPK)/extracellular signal-regulated kinase (ERK) and nuclear factor kappa-light-chain-enhancer of activated B cells (NF-kβ) signalling pathways, upregulation of transforming growth factor β (TGF-β), expression of pro-inflammatory cytokines [interleukin (IL)−1β, IL-6, IL-10, IL-12, IL-12/IL-23p40 and tumor necrotic factor-alpha (TNF-α)] and higher proliferation of macrophages, activation of 5′ adenosine monophosphate-activated protein kinase (AMPK), initiation of mitochondrial signalling cascade and death-inducing signalling complex formation, reduced vascular endothelial growth factor, cyclin D1, phosphorylated ribosomal protein S6 and matrix metalloproteinases expression, and blockage of programmed cell death ligand 1 (PD-L1)/programmed cell death protein 1 (PD-1) receptor binding [109,[124], [125], [126]] (Fig. 2). The low MW CS and oligochitosan apparently tend to induce cancer cell death by apoptosis. The higher MW CS exerts mixed modes of cell death via apoptosis and necrosis. The anti-cancer activity of CS may be modulated by its anti-oxidant and anti-inflammatory properties [123]. Lower MW CS exhibits a higher degree of anti-oxidant activity as shorter chains are characterized by weaker intramolecular hydrogen bonds [127]. The reactive groups of shorter chains are more accessible to scavenge cellular free radicals. The production of free radicals is closely linked to the inflammatory process [128]. Oligochitosan and CS have been identified to be able to reduce reactive oxygen species production, inhibit NF-kβ signaling pathway and regulate inflammatory enzymes such as nitric oxide synthase in the course of curbing the development of inflammation [129,130]. To further raise the anti-cancer activity, CS has been grafted with carboxymethyl, sulfate, sulfated benzaldehyde, triphenylphosphonium bromide, N-(2‑hydroxy)-propyl-3-trimethylammonium, O-palmitoyl, hexose and thymine moieties [109,[131], [132], [133]].

### 4.3 Nanocarrier design

CS and its derivatives are extensively exploited as drug carriers for cancer treatment and prevention [[134], [135], [136], [137], [138], [139], [140]]. They undergo micro-to-nanoscale transformation by spray drying, freeze drying, emulsification and microfluidic processes to enable particle surface decoration with targeting ligand, permeation enhancer, diagnostic element and other functional excipients, and reduced diameter that collectively promote cancer targeting and therapeutic action. “Drug-free” CS NPs, and through functional excipient decoration, are lately regarded as more cancer selective and cytotoxic than the neat CS. The tumor microenvironment is characterized by an acidic pH between 6 and 6.5 [141,142]. CS, with pKa values around 6.5 [143,144], is minimally ionized under normal physiological pH. It nonetheless undergoes protonation and solubilization into an uncoiled elongated linear configuration in acidic tumor microenvironments. Resonating with the surface-decorated targeting ligand, CS NPs can efficiently interact with the negatively charged membranes of cancer cells and endothelial cells of tumor vasculature which overexpress anionic surface moieties such as phospholipids, glycoproteins and proteoglycans to alter the cell membrane permeability and migrate intracellularly by endocytosis and diffusion to exert anti-cancer effects. Similar to CS, the CS NPs induce apoptosis/necrosis/autophagy, and mitigate angiogenic and metastasis tendency of cancer cells [109,145] (Fig. 2). Transforming CS into NPs has its mode of cancer cell death dictated largely by the physical size of NPs instead of the MW of CS. The larger NPs tend to exert cancer cell death via apoptosis. The smaller NPs mediate cancer cell death by necrosis. CS NPs smaller than 100 nm are relatively toxic, whereas particles larger than 400 nm have rapidly declining anti-cancer activity. The anti-cancer activity of CS NPs is influenced by their particle size more than that of zeta potential.

### 4.4 CRISPR-Cas9 CS-based nanocarrier

Delivering sgRNA and Cas9, in the form of a RNP complex, directly to the cells represents the simplest approach of all in gene editing [146], unlike sgRNA and Cas9 cassettes inserted in plasmids where a RNP complex must first be formed subsequent to cellular transcription and translation activities before gene editing at the target locus [147,148] or Cas9 mRNA translated *in vivo* to produce Cas9 and be presented simultaneously with sgRNA in the cellular target to edit genome [149,150]. CS NPs have been advocated as the delivery vehicle of CRISPR-Cas9 system in the form of plasmid or RNP complex with their foreseeable high processability, sustained-release characteristics, cellular transfection and retention efficiency and nucleus penetration capacity [147,149,151] (Table 2). With reference to cancer therapy, chemotherapeutics such as doxorubicin, paclitaxel and siRNA may be co-formulated with CRISPR-Cas9 and delivered concurrently to provide a synergistic anti-cancer action [[152], [153], [154]].

**Table 2:** CS-based nanocarrier for CRIPSR-Cas9.

NP type	CS attribute	Preparation method	Cargo load	Shape/Particle size/Zeta potential	Cell line/*in vivo*	Remark	Ref
FDTNPs	MW: 100 kDa Deacetylation degree: 85 %	Self-assembly	sgSurvivin Pdna; DOX	Round; 204 nm; 23 mV	4 T1 cells; Female BALB/c mice	*In vitro* and *in vivo* anti-tumor efficacies of FDTNP/DOX/sgSurvivin pDNA were superior to those of delivery of DOX or sgSurvivin pDNA alone. 91.0 % breast cancer regression were recorded in mice bearing breast tumor.	[152]
CS-PLGA NPs	MW: 110 kDa Deacetylation degree: 75 %−85 %	Double emulsion solvent evaporation method using homogenization	CRISPR-Cas9 plasmid; Cy3 siRNA	Round; 310.8 ± 1.7 nm; 32.5 ± 3.01 mV	HeLa cells, HEK-293T cells	Cy3 siRNA loaded on PLGA NPs showed an internalization of 4.6 % and MFI of 13.76 %, while that of CS-PLGA NP resulted in 89 % internalization and MFI of 67.95 % in HeLa cells. The *in vitro* GFP silencing assessed by anti-GFP siRNA and NPs resulted in 10 %–15 % silencing by PLGA NPs and 50–55 % silencing by CS-PLGA NPs in HeLa cells. In HEK-293T cells, the GFP silencing by CRISPR-Cas9 plasmid pX459 with CS-PLGA NPs was 80 %−83 %, while that of PLGA NPs was 11 % and of commercial Lipofectamine agent was 13 %.	[153]
CLPV NPs	MW: 110 kDa Deacetylation degree: 75 %–85 %	–	sg1-VEGFR2-Cas9 plasmid; Paclitaxel	Round; 241.6 ± 8.2 nm	HepG2 cells; Female Kunmingmice	The genome editing efficiency of sgVEGFR2-Cas9 in CLPV NPs achieved up to 38.6 % in HepG2 cells *in vitro* and 33.4 % *in vivo*. The CLPV NPs suppressed more than 60 % VEGFR2 protein expression in HepG2 cells *in vitro* and inhibited hepatoma progress by 70 % *in vivo*.	[154]
ACMC/TCMC-PSCaCO3 NPs	MW: 9.1 × 10^4^ g/mol Degree of methyl substitution: ≥ 80 %	Self-assembly through electrostatic attraction followed by coating	CRISPR-Cas9 plasmid	240 nm; −11 mV	HeLa cells, HEK-293T cells	The CRISPR-Cas9 plasmid was efficiently delivered to cancer cell nuclei to mediate genome editing, resulting in an efficacious knockout of CTNNB1 gene coding β-catenin.	[155]
Apt/HA-CS NPs	Low MW Deacetylation degree: 75 %−85 %	Self-assembly through electrostatic attraction followed by coating	CRISPR-Cas9 plasmid and sgRNA	Round; 141.4 nm; 19.8 mV	MCF-7 cells, HeLa cells, HEK293cells, SK-MES-1 cells; BALB/c nude mice	The introduction of AS1411 and HA coat to CS significantly improved the cellular uptake of CRISPR-Cas9 in tumor cells which brought about effective genome editing in cancer cells to knock out the FOXM1. Apt/HA-CS NPs dramatically down-regulated proteins involved in tumor progression (VEGF and Bcl-2) *in vitro.* SK-MES-1 tumor-bearing BALB/c nude mice exhibited significant tumor inhibition *in vivo*.	[156]
Endosomolytic peptide KALA and ACMC-decorated PS NPs	MW: 9.10 × 10^4^ g/mol Carboxylation degree: ≥ 80 %	Self-assembly	CRISPR-Cas9 plasmid	Round; 242.6 ± 4.2 nm; 6.5 ± 0.4 mV	MCF-7 cells	The NPs effectively delivered plasmid to cancer cell nuclei for genome editing and successfully knocked out CDK11 gene in targeted tumor cells, attributable to the cell penetrating and endosomal escape capability of KALA peptide as well as the tumor cell and nucleus targeting ability of AS1411 ligand.	[157]
CS-RFP NPs	MW: ∼1 kDa	Self-assembly	Cas9 RNPs-ssDNA; N-terminal of Cas9 was decorated with E-tag to supply negative charges	Round; 177 nm; −10 mV	HeLa cells	The CS-RFP NPs effectively entered the cancer cells by endocytosis and a fair amount of them can even directly reach the nucleus for genome editing. The CS-RFP NPs can alternatively serve as a robust fluorescent probe to track the delivery routine and transfection efficiency of bioactive macromolecules.	[158]
CaNPs/CaPNPs and CSNPs	–	Green synthesis	CRISPR plasmid	Semi-spherical; 130 nm; +27 mV	HEK-293 cells	Gene expression of EGFP was about 25 % in HEK-293 cells for CaP/CS NPs and more than 14 % for Ca/CaP/CS NPs.	[159]
CT/CRISPR polyplexes	-	Self-assembly by electrostatic attraction	CRISPR plasmid	Rough and dense surface; 69 nm; +39 mV	HEK-239 cells	CT/CRISPR polyplexes presented very low toxicity to HEK-293 cells (viability was more than 80 %) and gene expression of EGFP was about 28 % in HEK-293 cells.	[160]
mPEG-CS/DNA nanocomplexes	Low MW (15 kDa); CS conjugated with mPEG (5,000 kDa)	Nanocomplexation	pSpCas9–2A-GFP plasmid (pX458 vector)	165.7 ± 2.9 nm; 24.9 ± 0.9 mV	HEK293 cells; Cystic fibrosis-like mucus model (pH 6.5)	The nanocomplexes protected CRISPR-Cas9 from DNase I digestion and promoted its trans-mucus transport to raise the transfection efficiency.	[161]

Abbreviations: FDTNPs, Folic acid and 2-(diisopropylamino) ethyl methacrylate-double grafted trimethyl chitosan nanoparticles; sgSurvivin pDNA, Survivin CRISPR/Cas9 plasmid; DOX, doxorubicin; CS-PLGA NPs, Chitosan-coated poly (lactic-co-glycolic) acid nanoparticles; Cy3 siRNA, Cyanine3 siRNA; MFI, mean fluorescent intensity; CLPV NPs, Chitosan based lactobionic acid- paclitaxel-VEGFR2-nanoparticles; ACMC/TCMC-PSCaCO_3_ NPs, Protamine sulfate co-precipitated with CaCO_3_ coated with modified chitosan nanoparticles; TCMC, TAT peptide functionalized carboxymethyl chitosan; Apt/HA-CS NPs, Hyaluronic acid and AS1411 aptamer-coated chitosan nanoparticles; ACMC, AS1411-functionalized carboxymethyl chitosan; PS, protamine sulfate; CS-RFP NPs, Chitosan-coated red fluorescent protein NPs, RNPs; Ribonucleoproteins; ssDNA, single strand DNA; E-tag, Glutamate residues; CaNPs, nanoparticulate blend of calcium nanoparticles; CaPNPs, calcium phosphate nanoparticles; CSNPs, Chitosan nanoparticles; CT/CRISPR polyplexes, Chitosan tetrazole-based CRISPR polyplexes; mPEG, Polyethylene glycol monomethyl ether.

Cells: Breast cancer 4 T1 cells; Hepatoma HepG2 cells; Human breast adenocarcinoma MCF-7 cells; Human cervical cancer HeLa cells; Human embryonic kidney HEK-239 cells; Human lung cancer SK-MES-1 cells.

**Fig. 3:**
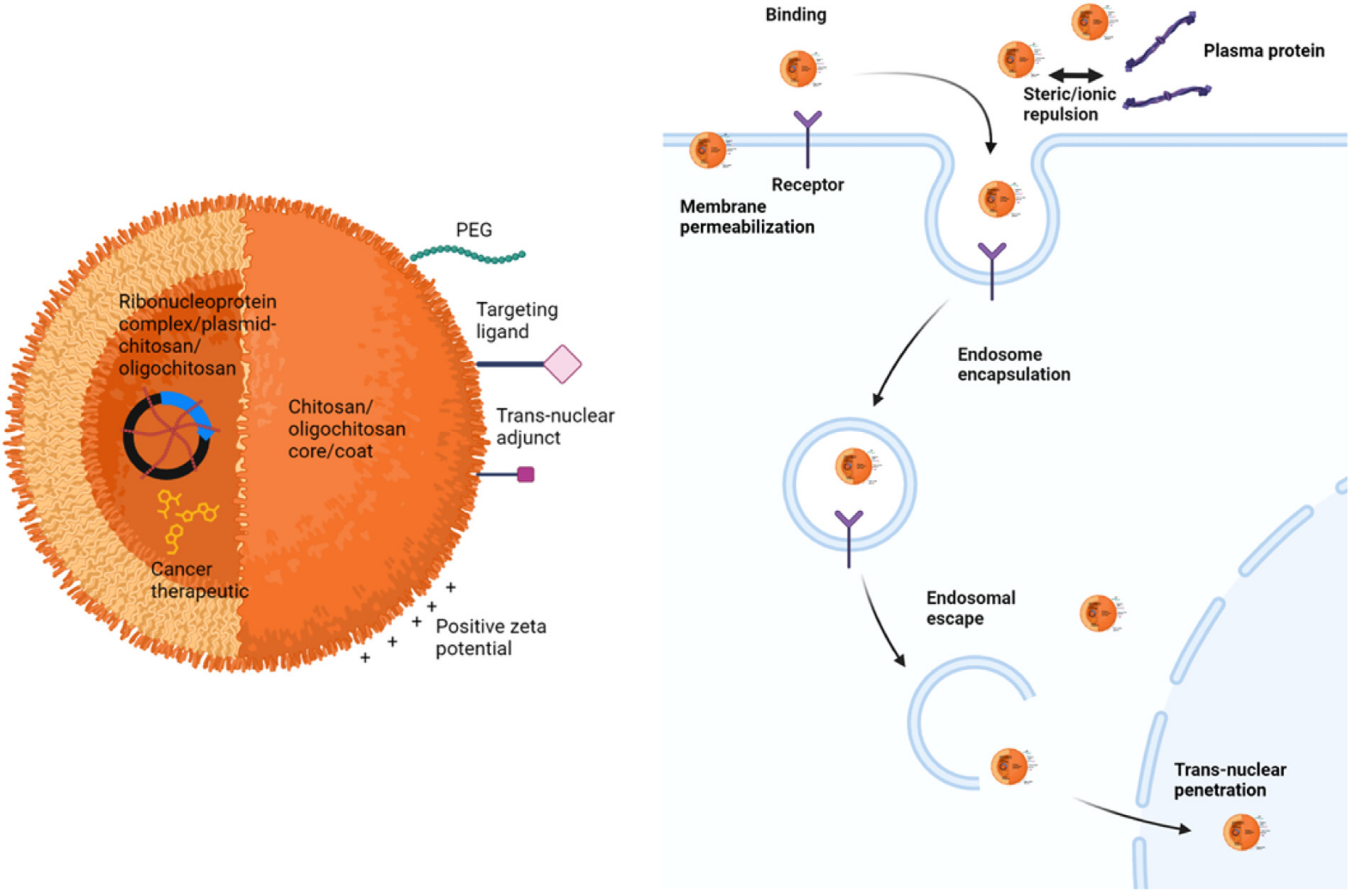
CS-based nanodelivery system of CRISPR-Cas 9.

CS, trimethyl CS, carboxymethyl CS, PEGylated CS, chitosan tetrazole and chitosan-lactobionic acid have been developed and employed as the matrix, coat or graft to the core (*i.e.* 2-(diisopropylamino) ethyl methacrylate, polylactic-co-glycolic acid), of the NPs acting as the carrier of CRISPR-Cas9 plasmid or RNP complex for cancer treatment [[152], [153], [154], [155], [156], [157], [158], [159], [160], [161], [162]] (Fig. 3; Table 2). The sizes of NPs are smaller than 250 nm in general, with positive or negative zeta potential developed on their surfaces as a function of formulation chemistry. Passive cancer targeting is attainable by means of enhanced permeability and retention effect [23]. The tumor growth progresses with the formation of large fenestrations at endothelial cell linings, in which pericyte attachment is loose, forming trans-vascular endothelial pores of sizes between 100 and 780 nm. These leaky vasculatures grant NPs accumulating by passive transport within the tumor tissues. NPs, having sizes between 20 nm and 500 nm, are more readily penetrating into cells via endocytosis or pinocytosis and this increases their transfection efficiency [58].

Cancer targeting of CRISPR-Cas9 by NPs can be further raised by the physiological stimuli-responsiveness of CS and its associated excipients such as aptamer AS1411, HA, β-galactose containing lactobionic acid and folic acid [152,[154], [155], [156], [157]] (Fig. 3). CS ionizes into positively charged species largely at the acidic tumor microenvironment and NP of such are preferentially bound to the negatively charged cancer mucus and mucosa over the normal tissue [23,163]. Aptamer AS1411, HA, lactobionic acid and folic acid on the other hand serve as the targeting ligand of CRISPR-Cas9 loaded NPs and raise their interaction affinity to cancer cells via binding to nucleolin, cluster of differentiation 44 (CD44), asialoglycoprotein and folate receptors overexpressed on tumors [152,154,156]. Alternatively, the CRISPR-Cas9 can be immobilized on magnetic peptide-imprinted CS NPs where its delivery specificity can be modulated by external magnetic field [162].

To succeed both passive and active cancer targeting, it is imperative that the CRISPR-Cas9 is encapsulated with a high level of physicochemical stability in the CS-based NPs with minimal premature leaching, degradation, reticuloendothelial clearance, protein corona formation and particulate aggregation. CS is characterized by a high density of cationic charges. It can be used to condense plasmid of CRISPR into nanocomplexes and the level of condensation can be further raised by crosslinking the CS with tripolyphosphate counterions or optimizing the complexation conditions: CS MW and degree of deacetylation, complexation pH and N/P ratio of CS to nucleotide [159,164,165]. The favorable complexation of CS with nucleotides promotes encapsulation, minimizes leaching and degradation by endonucleases found in physiological fluids, reduces the negative charges of plasmid and induces the formation of smaller particles that facilitate their interaction with cancer cell membrane and uptake [[166], [167], [168], [169]]. With reference to CS-RNA polyplex formation, stable and transfected complexes are produced using CS with a MW of 10 to 140 kDa [170,171].

PEG has been introduced as a part of the CS NPs formulation of CRISPR-Cas9 [161] (Fig. 3). PEG, decorated on the surfaces of the NPs, could act as a coat barrier to protect the gene cargo from leaching and degradation [172]. The PEGylated NPs are relatively hydrophilic, stable in the systemic environment and undergo reduced reticuloendothelial clearance which allow them to have a prolonged circulation to exert passive and active cancer targeting [23,93]. PEGylation of NPs provides steric hindrance and a strong negative surface charge attributed to the carboxy terminal of PEG [49,173]. It could reduce the aggregation tendency of NPs and their interaction with the surrounding proteins to form protein corona that itself leads to further particulate aggregation and risks of embolism. The hydrophilic nature of PEG favors its hydrogen bonding with the water molecules into a hydration shell which hinders NPs from interacting with opsonin proteins and mitigates their premature clearance by mononuclear phagocyte system [23]. PEGylation of monomethyl ether PEG to the hydroxyl group of CS NPs has been reported to increase the transfection efficiency of CRISPR-Cas9 [161]. This is aptly explained by PEGylated NPs having reduced surface charge which facilitates their transport across the thick mucus, a prior barrier before the target site. Overall, PEGylation can prolong the biological survival and promote the trans-mucus diffusion of NPs. Nonetheless, the hydrophilic PEG could reduce the cancer cell uptake of these NPs [174]. Introducing surface negative charges onto the NPs via carboxymethylation of CS could bring about similar benefits as PEGylation in reducing protein corona formation and particle aggregation [23]. This approach however may not be able to raise the cancer cellular uptake propensity of NPs due to electrostatic repulsion developed between the negatively charged carboxymethyl CS NPs and cancer cell membrane.

Succeeding CS-based NPs transit from the site of administration, through blood or bodily fluids, to cancer target per se is apparently inadequate to fully meet the delivery performance of CRISPR-Cas9. At the cancer site of action, these NPs must be able to overcome mucus, mucosa and/or nuclear membrane barrier of the cancer cells bridging the host DNA with CRISPR-Cas9 load. PEGylation aids trans-mucus transport of CS NPs [161]. The nanoparticulate CS can act as a permeation enhancer through fluidizing the cancer cell membrane and opening the intercellular tight junction to facilitate the cellular uptake of NPs [23,137] (Fig. 3). With reference to cancer therapy, CS has been thiolated or methylated to develop permanent positive charges in facilitation of its binding to cancer cells via disulphide bonds with mucus glycoproteins (*e.*g*.* mucin) and ionic interaction between the amine moiety in CS and sulfonic acid substructures of mucus in addition to hydrogen bonding between the hydroxyl and amino groups of CS and mucin. The positive charge of CS aids reversible opening of the tight junctions by activating the protein kinase C pathway [50] and disrupting the junctional proteins zonula occudens-1 and occludin [23]. CS has been functionalized with HIV-derived TAT peptide (GRKKRRQRRR) and cell penetrating compounds [23,175,176]. Combination of cell penetrating ligand and methylation/thiolation can further raise the cancer cell membrane permeabilization for effective NP uptake. The intracellularly entrapped CS NPs in endosomes, subsequent to endocytic process, could fuse with lysosomes and subject CRISPR-Cas9 to risks of enzymatic hydrolysis [144,163,177,178]. Amino-rich CS can attract protons along with water and chloride resulting in endosomes undergoing osmotic swelling and inducing endosomal escape of NPs from lysosomal digestion under the proton sponge effect (Fig. 3). Its protective role on genetic materials can be enhanced by hybridization of CS NPs with fusogenic peptides or pH-sensitive neutral lipids such as di-oleoyl-phosphatidyl-ethanolamine that improve fusion of carriers with endosomal membrane for escape into the cytoplasmic domain [179]. Transporting CS NPs or CRISPR-Cas9 from cytoplasm into nucleus is expected to be hindered by double-layered membrane perforated by protein channels (nuclear pore complex) characterized by a small nuclear pore size (∼10 nm) [180]. HIV-TAT and cyclic TAT have been proposed as the adjunct ligand to promote nuclear delivery [23,155,181,182] (Fig. 3). The Cas 9 protein has been altered by inserting three repeated nuclear localization signals to provide nuclear targeting and additional negative charges for electrostatically binding to CS into small nanocomplexes where further physicochemical modification into larger NPs with reduced negative surface charges has them remained confined to the cellular nucleus [158] Protamine has also been used as adjunct material to encapsulate CRISPR-Cas9 plasmid to promote membrane transport as well as nuclear translocation [155].

Through excipient decoration and particle design, a minimal of 25 % to more than 70 % of cancer cells can be transfected with CRISPR-Cas9 by the CS-based NPs with reference to colon cancer, breast cancer, lung cancer and embryonic kidney cell populations [153,154,156,158,160]. As high as 80 % of genes can be silenced and 70 % of cancer growth eg. hepatoma carcinoma can be halted with over 60 % reduction in the expression of vascular endothelial growth factor receptor 2.

## 5 Challenges and critical insights

CS, of oligomeric or low MW attribute, despite popularly used as the carrier material of cancer chemotherapeutics and genetic drugs, has only been adopted in a limited number of studies pertaining to CRISPR-Cas9 delivery (Table 2). It is advantageous to adopt oligomeric or low MW CS as nanocarrier as they were inherently cancer cytotoxic and tend to induce cancer apoptosis instead of necrosis [109]. Co-delivery of doxorubicin and DNA plasmid into MCF-7 breast cancer cells is characterized by 52.2 % of cells carrying doxorubicin solely and 42.7 % of cells engulfing both doxorubicin and plasmid [183]. Such disparity may be ascribed to doxorubicin, as a smaller therapeutic molecule, is absorbed into cancer cells in the form of freely release molecules in the extracellular fluid as well as nanoencapsulated species admixed with plasmid. It infers that a single treatment of cancer by CRISPR-Cas9 may not benefit all cells. Developing an effective dosage regimen *in vivo* against cancer growth and relapse is essential to realize the future clinical translation of CRISPR-Cas9 for cancer treatment.

NPs are regarded as an ideal drug delivery vehicle for cancer treatment under the precision nanomedicine platform. The practice of precision medicine adopts omics-led drug molecule-cancer molecular target matching approach in therapeutic selection [47]. The introduction of nanotechnology into precision medicine merely promotes passive and active targeting of the selected therapeutic, with the aim to further improve drug efficacy and minimize drug adverse effects. Active targeting of cancer involves the use of targeting ligands in the development of nanomedicine. Thus far, the choice of targeting ligand is not identified through precise characterization of the overexpressed cancer receptor which can be unstable and varies with tumor growth rate, metastasis, fasting, concurrent diseases and drugs used. The expression of estrogen receptor, progesterone receptor and human epidermal growth factor receptor-2 may be reduced by human cytomegalovirus infection in primary breast cancer and brain metastases. Receptor expression may be up- or down-regulated by estradiol, tamoxifen, medroxyprogesterone acetate and other chemicals. The drugs may induce methylation and acetylation of gene leading to the epigenetic events that end with receptor overexpression or *vice versa*. The primary tumor is characterized by a higher receptor population density as it grows larger with time, with cancer and stromal cells exhibiting different distribution for the same receptor type. Making an appropriate choice of targeting ligand for cancer nanomedicine development requires an in-depth understanding of cancer receptor profiles of patients and matching the targeted receptor with a truly functional targeting ligand to avoid incurring off-target delivery outcomes. As such, omics profiling of cancer patients should not merely provide the molecular target data, but include the cancer receptor distribution and related details.

PEG is a well-established stealth material used in nanomedicine formulation. It is metabolized under the catalysis of alcohol dehydrogenase into aldehydes and acids [47]. Approximately 5 %–10 % of English, 20 % of Swiss and 85 % of Japanese populations express atypical alcohol dehydrogenase and metabolize PEG at a faster rate than those with native enzyme. The PEGylated CS NPs of CRISPR-Cas9 may undergo an accelerated cargo leaching and clearance due to premature biodegradation of the surface PEG. This in turn is expected to negate their biological performance as a cancer therapeutic. Such complexities in induction and inhibition behaviors of the metabolizing enzymes are similarly found under the influences of drugs, diseases, diet and smoking. Further, the PEGylated CS NPs of CRISPR-Cas9 risks binding and removal by anti-PEG immunoglobulin [47,184]. A higher plasma concentration of anti-PEG immunoglobulin M is associated with SNP rs12590237 genetic polymorphism in the variable segment of the immunoglobulin heavy chain (IGH). The genetic make-up of cancer patients pertaining to their antibody and metabolizing enzyme profiles needs an in-depth assessment to enable right excipient selection in nanomedicine development and avoid premature digestion of the excipients prior fulfilling their roles in drug delivery.

CS is a cationic polysaccharide. Cationic and hydrophobic NPs are prone to the formation of protein corona in site of administration such as gastrointestinal tract and lung as well as in the systemic circulation through binding to the surrounding protein thus leading to surface charge depression and particle aggregation [49]. The negatively charged NPs may be predisposed to protein corona formation and aggregation as biological proteins such as fibrinogen have positive charge domain that could interact with these NPs [35]. PEGylation is one approach that has been adapted to mitigate protein corona formation and aggregation of ionic NPs of CRISPR-Cas9 [185]. Incomplete nanoparticulate surface coverage by PEG however could defeat the purpose of PEGylation [49]. Use of long PEG chains may risk particle aggregation due to increased cohesiveness and reduce cell transfection efficiency [186].

Developing CS NPs of CRISPR-Cas9 with hydrophilic and steric characters is imperative to enable them to succeed in administration site-blood-target axis transit. Hydrophobic and cationic characters are essential to promote NPs-cancer cell membrane interaction and tight junction opening which grant cancer cell uptake of these matrices. Adequate surface projection of the targeting ligand of NPs to intimate the cancer receptor is the pre-requisite of cancer cell targeting. The cationic property of CS is essential to incur proton sponge effect intracellularly in protection of the cargo load of the NPs from lysosomal digestion. The NPs or complexes must be sufficiently small to penetrate the nuclear pores in delivery of RNP to the cell nucleus. Small nano geometry remains a paramount criterion to drive CRISPR-Cas9 targeting at cellular and sub-cellular levels. The surface chemistry of NPs nonetheless requires hydrophilicity/hydrophobicity and ionic transformation to enable them to transport from the site of administration to the intracellular target. The core or/and coat of NPs requires excipient switching via labile bond lysis or be chemically reacted to the external stimuli such as pH to confer the intended surface physicochemical property for cellular transport.

Cancer nanomedicine design for CRISPR-Cas9 delivery involves a multitude of efforts by pharmaceutical scientists, chemists, medical professionals, material scientists, biotechnologists and laboratory analysts. The initial laboratory success may be hampered by the subsequent manufacturing challenges. NPs can be processed and harvested using spray drying, freeze drying and nanoemulsification techniques [187]. Owing to small fluffy feature and electrostatically unstable, the production of NPs may be as low as 20 % and irreproducible. The size and surface composition of the NPs are susceptible to changes with time and are affected by the production and storage environment, following aggregation, dissolution, sorption, and/or corrosion process. Such particulate alterations could deviate the intended biological fate of the nanomedicine due to poor delivery performances. Soft agglomerates, physical blend and compact have been advocated as the carrier of NPs to enable the drug load to survive the harsh pH/enzymatic environment of the administration site prior reaching the target site or reduce the aggregative behavior of NPs without them growing into sizes exceeding the diameter of the delivery pathway. Intracellularly, the accessibility of CRISPR-Cas9 to DNA sequence can be modulated via tethering the Cas9 nuclease to histone modifiers and proteins involved in altering DNA methylation and silencing the distal regulatory element Kruppel-associated box repressor [188,189]. Will CS-based NPs as CRISPR-Cas 9 carrier be useful to suppress the epigenetic control of gene expression in facilitation of gene editing?

## Conflict of interest

None.
